# An exploration of the determinants of risk formulation, care plan and disposition among older adults in the Ontario forensic psychiatry system: implication for practice

**DOI:** 10.1017/S1092852925100242

**Published:** 2025-05-15

**Authors:** Mark M. Kaggwa, Joan Abaatyo, Arianna Davids, Angela Li, Rebecca Marsh, Precious Agboinghale, John M. W. Bradford, Gary A. Chaimowitz, Andrew T. Olagunju

**Affiliations:** 1 Department of Psychiatry and Behavioral Neurosciences, McMaster University/St Joseph’s Healthcare Hamilton, Hamilton, ON, Canada.; 2 Forensic Psychiatry Program, St Joseph’s Healthcare Hamilton, Hamilton, ON, Canada; 3 Department of Psychiatry, Uganda Christian University, Kampala, Uganda; 4 Department of Psychiatry, King Ceasor University, Kampala, Uganda; 5 Michael G DeGroote School of Medicine, McMaster University , Hamilton, ON, Canada.; 6 Division of Forensic Psychiatry, University of Ottawa, Ottawa, ON, Canada; 7 Department of Psychiatry and Behavioral Sciences, University of Oklahoma College of Medicine, Oklahoma City, OK, USA; 8 Discipline of Psychiatry, The University of Adelaide, Adelaide SA, Australia; 9 Department of Clinical Services and Research, Federal Neuropsychiatric Hospital, Calabar, Cross River State, Nigeria

**Keywords:** Forensic psychiatry, geriatrics, index offense, not-criminally responsible, risk, substance use, unfit-to-stand trial

## Abstract

**Background:**

Advances in medicine have led to an improvement in life expectancy, thus increasing the population of older individuals within the criminal justice system. This study investigates the determinants of risk formulation, care plan, and disposition among older adult forensic patients (OAFP) in Ontario, Canada.

**Methods:**

This retrospective analysis utilized the Ontario Review Board database, focusing on 161 OAFP, aged 55 years and older. Hierarchical regression was used to analyze the relationship between changes in risk and six blocks of variables: sociodemographic characteristics (Block 1), circumstances during the index offense (Block 2), current clinical profile (Block 3), past psychiatric history and behavioral patterns (Block 4), criminal history and legal status (Block 5), and recent violent events (Block 6).

**Results:**

The median age of patients was 61 years (IQR 58–67), with 83.4% being male. Schizophrenia was the most common diagnosis (68.3%), and 9.3% had neurocognitive disorders. The model with six blocks of factors explained 92% of the variability in risk change. Models 2 (blocks 1 and 2) and 4 (blocks 1–4) were statistically significant, explaining 34% (p = 0.010) and 22% (p = 0.018) of the variance in the change in risk of threat to public safety, respectively. OAFP with a significant risk to public safety were more likely to be inpatients and less likely intoxicated during their index offense.

**Conclusion:**

Resources, policies, and a supervised model of care to curtail behavioral risks are relevant to the care of OAFP. Innovative risk management models for OAFP are indicated.

## Introduction

Advances in technology and medicine have led to an improvement in life expectancy, thus increasing the population of older individuals aged 50 years and older, including those within the criminal justice system.^1^ Understanding the unique attributes and needs of older adults involved in the criminal justice system is increasingly becoming important in forensic psychiatric contexts to ensure an accurate formulation of their needs or risk to public safety and develop effective management, albeit these needs can easily be overlooked.^2^^-^^5^ More often, patients in the forensic systems are treated and rehabilitated for a reasonable period to ensure that they are released at a point when their risk to the public is managed and are less likely to reoffend.^3^^,^^5^ While a considerable proportion of individuals in the criminal justice system in Ontario are older adults at the time of their index offense, over half of these older adults entered the correctional system at a younger age, especially before 50 years, and transitioned into older adults following a prolonged time in detention.^6^ Such lengthy stays in the criminal justice system are often associated with noticeable changes in health and psychosocial status, including those that are due to aging.^7^^,^^8^ For instance, older individuals within the forensic-correctional system are more likely to experience cognitive decline, mental health disorders, and complex medical conditions, all of which can heighten their needs and the unpredictability of their behavior.^5^^,^^6^^,^^8^^,^^9^ With age, there may be the cumulative effect of untreated psychiatric issues or a history of aggression, which, when coupled with conditions like dementia or mood disorders, can heighten the vulnerability to behavioral problems that are akin to poor judgment, erratic or even violent behaviors.^5^^,^^9^^,^^10^ Despite these risks, older individuals in the criminal justice system may not receive adequate attention in terms of their needs to address the potential risk for harmful behavior or re-offending, partly because aging is thought to reduce the likelihood of offending tendencies.^9^^,^^10^ However, their vulnerability, combined with a possible history of untreated or poorly managed psychiatric or medical conditions and other psychosocial life events, makes older adult forensic patients (OAFP) a population that requires specialized care, added support, and comprehensive risk mitigation strategies.^4^ This is partly supported by the level of recidivism associated with older populations, especially with over 50% of older individuals above 50 years in Ontario’s correctional system detained for re-offending.^6^.

In the forensic psychiatric context, assessing the risk an individual poses to public safety is a key aspect of clinical practice that informs the decision process for disposition, community reintegration, risk mitigation strategy, and resource allocation to address psychosocial needs.^5^^,^^11^^,^^12^ This risk assessment involves a detailed evaluation of clinical, behavioral, and criminal histories to formulate an individual’s risk profile, with the goal of identifying factors that predict a heightened risk or threat to public safety and what care plan is needed to mitigate risk.^5^^,^^11^ Traditionally, factors such as psychiatric diagnoses, demographic characteristics, and previous risky behaviors are considered when evaluating potential threats to public safety and planning appropriate care.^13^ However, certain characteristics, such as inpatient clinical status, younger age at first psychiatric admission, or previous history of violence, have been identified as particularly strong predictors of future risk and trajectory of their care.^14^^,^^15^

While the majority of research on risk assessment in forensic psychiatry has focused on younger populations, there is a growing recognition of the need to examine older adults more closely to help understand the complexity of their needs and inform nuanced approaches to their care.^3^^,^^5^^,^^8^^,^^16^ With the trend in the aging of the world’s population, the burden of age-related mental and physical health issues (e.g., Alzheimer’s disease, neurodegenerative, dementia, delirium, and other organic mental disorders), and their associated behavioral and psychological problems (e.g., sexual disinhibition, frontal lobe syndrome, aggression, and agitation) among affected older adults is expected to rise.^17^^-^^19^ Taken together, the increasing burden of these age-related behavioral problems underscores the importance of understanding the needs of older adult offenders and what is needed to mitigate the risk posed to the affected individuals, their families, and public safety. While recognizing a scarcity of research on older forensic psychiatric populations, a better understanding of the unique challenges and needs associated with aging can promote accurate assessment, inform appropriate strategies, help clinicians to better manage any risk issues, and enhance community re-integration of OAFP.^3^ This study aims to address the gap in research by examining the predictors of change in public threat and risk among older individuals in forensic psychiatric care, providing insights that can inform the care of older adults, clinical practice, and public safety measures.

## Methods

### Study design

This is a retrospective study involving older individuals aged 55 years and older in the Ontario Review Board (ORB) database. All patients under the auspices of the ORB underwent forensic psychiatric assessments to support the legal determination of their forensic status. These patients are either inpatients or outpatients, depending on their disposition and clinical status. Annual progress is documented and presented to the ORB for each patient.^20^^-^^22^

The database was created from the information curated from the annual ORB reports for the reporting years of 2014 and 2015. The data from this ORB database have been used and described in other published papers.^20^^-^^22^ The current analysis only included older individuals whose risk profile for public safety was captured during the reporting years (2014/15) under reference and the previous year.

### Study variables

#### Outcome variable

**Change in Risk of Threat to the Public:** This variable was determined by calculating the difference between the risk levels in the reference study years (2014 and 2015) and previous reporting years. This outcome is very crucial and fundamental to the core mandate of the forensic psychiatry system because according to Section 672.54 of the Criminal Code of Canada, an individual must pose a significant threat to the public to remain under the care of the forensic psychiatry system.^23^ A significant threat to public safety is defined as a risk of serious physical or psychological harm to members of the public, including any victim or witness of the offense, or any person under the age of 18, resulting from conduct that is criminal in nature but not necessarily violent.

Each year, the risk each patient poses to public safety is accessed and communicated in the ORB reports that are presented at the annual hearing of the ORB. Following an integrated multi-source comprehensive risk assessment, individuals in the forensic system are categorized and coded based on their risk profile as follows: **None:** Does not pose a significant risk to the public. **Moderate:** Poses a moderate threat to public safety **High:** Poses a significant threat to public safety.

In this study, we operationalized these different levels of risk with numerical values, such that those with no significant risk were coded as zero (0), one (1) for a moderate, and two (2) for a high significant risk for public safety. The difference between the risk levels in the reporting year under study and previous years was calculated to derive a continuous outcome variable labeled as the change in risk level for a threat to public safety. This variable can have positive or negative values, reflecting increases or decreases in risk levels. This change in risk level was used as the main outcome variable in the current study.

#### Model categories and variables

We considered six blocks of plausible variables within the dataset to explain the changes in the risk to public safety. These panels of risk factors are informed by factors considered essential in ORB reports, available literature regarding risk prediction, and input from clinicians with expertise in forensic psychiatry risk management.^5^^,^^24^^-^^26^ These blocks included sociodemographic characteristics (Block 1), circumstances during the index offense-the crime or offense that led to their forensic status (Block 2), current clinical profile (Block 3), psychiatric history and behavioral patterns (Block 4), criminal history and legal status (Block 5), and recent violent events (Block 5).

##### Sociodemographic characteristics

This foundational block includes critical variables such as age, gender, employment status, level of education, and country of birth. These variables provide a baseline understanding of the individual’s background.

##### Circumstances during the index offense

This block captures the context of the index offense, including the age of the individual at the time of the offense, intoxication status (yes/no), the nature of the offense (violent, sexual, non-violent), victim characteristics (gender and age), the relationship to the victim (known/unknown), and the severity of injury inflicted (none, minor, hospitalization, death).

##### Current clinical profile

This block encompasses variables that reflect the individual’s current mental health status for the period under review (2014 and 2015), including primary diagnosis, presence of comorbid psychiatric conditions (yes/no), outpatient or inpatient status, length of stay within the forensic psychiatry system (in years), and capacity to consent to treatment (yes/no). Assessments included in the ORB reports were conducted by trained and certified forensic psychiatrists in Canada, and diagnoses were made according to the Diagnostic and Statistical Manual of Mental Disorders (DSM) criteria. Additionally, a battery of psychological tools is administered by certified clinical psychologists to support diagnostic and capacity assessments.

##### Past psychiatric history and behavioral patterns

This block includes comprehensive variables such as age at first admission for mental health concerns, history of substance use, history of self-harming behaviors before the reporting year, previous instances of absconding, past risk behaviors (e.g., physical violence toward objects, people, inappropriate sexual behaviors), previous incapacity to consent to treatment, compliance with treatment, and historical response to treatment.

##### Criminal history and legal status

This block details the individual’s criminal background, including the number of previous convictions, history of absconding (yes/no), previous number of incarcerations, and current Ontario Review Board (ORB) status (not criminally responsible on account of mental illness [NCR] or unfit to stand trial [UST]).

##### Recent violent events

This final block assesses a recent involvement in a violent behavior/incident (as a perpetrator) or self-harm during the reporting year, including verbal aggression, harm to objects, harm to others, inappropriate sexual behaviors, and self-harm.

### Data analysis

The data were analyzed using STATA version 17. Categorical variables were presented as frequencies and percentages, while the continuous variables (e.g., age during the reporting year, age at index offense, length of stay within the forensic system, among others) were described using either the mean and standard deviation (SD) or the median and interquartile range (IQR). Inferential statistics was assessed using analysis of variance (ANOVA) and student *t*-test. Changes in public threat risk were treated as a continuous variable. Hierarchical multivariate linear regression was conducted to identify predictors of increased or decreased public threat risk using the Stata command “nestreg, waldtable.” A significance level of less than 5% was considered for a 95% confidence interval.

## Results

The study included 161 individuals aged 55 years and older who had information on previous and reporting years risk levels captured to allow an estimate of changes in risk levels. The median age of older participants in the forensic psychiatry program in Ontario during the reporting year was 61 years, with an interquartile range (IQR) of 58–67. Most individuals were between 55 and 65 years old (65.2%) and were predominantly males (81.4%, n = 131). The primary diagnosis for the majority was schizophrenia (68.3%) followed by bipolar disorder (9.3%) and 9.3% had a diagnosis of a neurocognitive disorder. None of the included individuals had a violent index offense. The majority (80.8%, n = 130) posed a highly significant threat to public safety during the reporting year (2014 and 2015), although this percentage was lower than the 87.5% reported in the previous year (see Table 1).

**Table 1.** Distribution of Study Characteristics

Variable	n (%)	Change in risk	r/t/F test (p-value)
***Categorization of risk levels for the reporting versus previous year***			
**Threat to public for previous year (n = 161)**			
None	8 (5.0)		
Moderate	12 (7.5)		
High	141 (87.5)		
**Threat to public for the reporting year (n = 161)**			
None	17 (10.6)		
Moderate	14 (8.7)		
High	130 (80.8)		
***Sociodemographic characteristic***			
**Age** *[median (IQR)] (years)*	61 (58–67)	−0.01	0.869
**Age categories (years)**			
55–65	105 (65.2)	−0.1 (0.6)	0.46 (0.633)
65–75	43 (26.7)	−0.1 (0.4)	
>75	13 (8.1)	0 (0)	
**Gender**			
Female	30 (18.6)	−9.1 (0.7)	0.01 (0.914)
Male	131 (81.4)	−0.1 (0.4)	
**Employment status (n = 156)**			
Employed	7 (4.5)	−0.1 (0.4)	0.01 (0.939)
Unemployed	149 (95.5)	−0.1 (0.5)	
**Level of education (n = 148)**			
Below secondary	86 (58.1)	−0.2 (0.5)	0.01 (0.943)
Secondary to post-secondary	62 (41.9)	−0.2 (0.4)	
**Citizenship (n = 139)**			
Canadian born	90 (64.8)	−0.1 (0.4)	0.05 (0.821)
Not Canadian born	49 (35.2)	−0.1 (0.5)	
***Circumstances during the index offense***			
**Age at index offense** *[mean (SD)]*	52 (11.8)	−0.08	0.311
**Intoxication at index offense (n = 158)**			
No	140 (88.6)	−0.1 (0.5)	0.70 (0.403)
Yes	18 (11.4)	−0.2 (0.5)	
**Use of weapon at index offense (156)**			
No	98 (62.8)	−0.1 (0.5)	0.67 (0.413)
Yes	58 (37.2)	−0.2 (0.5)	
**Victim’s gender (n = 119)**			
Male	48 (40.3)	−0.1 (0.6)	0.05 (0.65)
Female	71 (59.7)	−0.1 (0.4)	
**Relationship with victim (n = 126)**			
Known to patient	84 (66.7)	−0.2 (0.5)	3.35 (0.07)
Unknown to patient	42 (33.3)	0 (0.4)	
**Level of injury to victim (n = 124)**			
Minor/none	86 (69.4)	−0.1 (0.5)	0.43 (0.648)
Requiring hospitalization	19 (15.3)	−0.2 (0.6)	
Resulting in death	19 (15.3)	−0.1 (0.5)	
**Nature of index offence**			
Sexual	19 (11.8)	−0.1 (0.2)	0.42 (0.515)
Non-violent offence	142 (88.2)	−0.1 (0.5)	
***Current clinical profile***			
**Primary diagnosis**			
Schizophrenia and psychotic spectrum disorder	110 (68.3)	−0.1 (0.6)	0.30 (0.965)
Bipolar disorder	15 (9.3)	−0.2 (0.6)	
Neurocognitive disorder	15 (9.3)	0 (0)	
*Others*			
Substance use disorder	3 (1.9)	0 (0)	
Major depressive disorder	3 (1.9)	0 (0)	
Neurodevelopmental disorder	3 (1.9)	0 (0)	
Personality disorder	5 (3.1)	0 (0)	
Sexual disorder (paraphilia)	6 (3.7)	−0.2 (0.4)	
Traumatic brain injury	1 (0.6)	0 (0)	
**Comorbid mental illness**			
No	55 (34.2)	0.0 (0.5)	2.51 (0.12)
Yes	106 (65.8)	−0.2 (0.5)	
**Clinical status**			
Out-patient	76 (47.2)	−0.3 (0.8)	**11.41 (<0.001)**
In-patient	85 (52.8)	0 (0)	
**Length in the forensic psychiatric system (years) [median (IQR)]**	7 (3–14)	0.04	0.643
**Capacity to consent for treatment (n = 152)**			
No	77 (50.7)	−0.2 (0.6)	**4.64 (0.033)**
Yes	75 (49.3)	0 (0.4)	
**Treatment adherence (n = 143)**			
Adherent	131 (91.6)	−0.2 (0.6)	0.81 (0.369)
Non-adherent	12 (8.4)	0 (0)	
***Past psychiatric history and behavioral patterns***			
**Age at first admission [median (IQR)]**	33 (23–54)	−0.12	0.115
**History of substance abuse (n = 214)**			
Yes, alcohol and other illicit drugs	38 (24.0)	−0.1 (0.6)	0.43 (0.735)
Yes, alcohol only	9 (5.7)	−0.1 (0.3)	
Yes, other illicit drugs only	5 (3.2)	−0.2 (0.7)	
No	106 (67.1)	−0.2 (0.5)	
**History of self-harm (n = 156)**			
No	140 (89.7)	−0.1 (0.5)	0.99 (0.32)
Yes	16 (10.3)	−0.3 (0.7)	
**History of absconding**			
No	116 (72.0)	−0.1 (0.5)	0.02 (0.888)
Yes	45 (28.0)	−0.1 (0.6)	
**Past violent events to objects (n = 157)**			
No	122 (77.7)	−0.1 (05)	0.89 (0.346)
Yes	35 (22.3)	−0.2 (0.6)	
**Past violent events to others (157)**			
No	73 (46.5)	−0.1 (0.5)	0.05 0.828)
Yes	84 (53.5)	−0.1 (0.5)	
**Past sexually inappropriate behavior (n = 157)**			
No	106 (67.5)	−0.1 (0.6)	0.03 (0.870)
Yes	51 (32.5)	−0.1 (0.4)	
**Previous incapacity to consent for treatment (n = 146)**			
No	61 (41.8)	−0.2 (0.6)	0.27 (0.606)
Yes	85 (58.2)	−0.1 (0.5)	
**Previous poor treatment compliance (n = 145)**			
No	31 (21.4)	−0.1 (0.4)	0.62 (0.432)
Yes	114 (78.6)	−0.2 (0.6)	
**Previous response to psychiatric treatment (n = 132)**			
Good	25 (18.9)	−0.2 (0.7)	1.36 (0.25)
Poor	107 (81.1)	−0.1 (0.5)	
***Criminal history and legal status***			
**Number of previous convictions [median (IQR)]**	1 (0–7)	−0.07	0.358
**Previous incarceration (n = 150)**			
No	77 (51.3)	−0.1 (0.4)	0.67 (0.415)
Yes	73 (48.7)	−0.2 (0.5)	
**Review board status**			
Unfit to stand trial (UST)	23 (14.3)	−0.1 (0.3)	0.14 (0.707)
Not criminally responsible (NCR)	138 (85.7)	−0.1 (0.5)	
***Recent violent events***			
**Recent incidence of harm to objects (n = 159)**			
No	146 (91.8)	−0.1 (0.0)	−0.92 (0.357)
Yes	13 (8.2)	0 (0)	
**Recent incidences of harm to self**			
No	157 (98.7)	−0.1 (0.0)	−0.35(0.728)
Yes	2 (1.3)	0 (0)	
**Recent incidences of harm to others**			
No	132 (83.0)	−0.2 (0.1)	−1.40 (0.162)
Yes	27 (17.0)	0 (0)	
**Recent incidences of sexually inappropriate behavior**			
No	133 (83.7)	−0.2 (0.1)	−1.37 (0.172)
Yes	26 (16.4)	0 (0)	
**Recent incidences of verbal aggression**			
No	112 (70.4)	−0.2 (0.1)	−2.0 (0.045)
Yes	47 (29.6)	0 (0)	

Abbreviation: IQR, interquartile range; n, frequency; SD, standard deviation.

### Changes in the risk of threat to the public

The change in the risk of threat to the public was assessed for 161 individuals who had information on their risk levels for the previous and reporting year assessed. Outpatient clinical status (p-value <0.001) and a lack of capacity to consent to treatment (p-value = 0.033) were significantly correlated with a decreased risk of threat to the public statistically (see Table 1).

### Predictive models for factors associated with change in risk to public safety

Table 2 presents a multiple hierarchical linear regression analysis with six models predicting the changes in the risk of posing a significant threat to public safety among 161 individuals with data on previous and current risks during the period under study.

**Table 2.** Predictive Models of Increase in Risk of Threat to Public

Variable (n = 161)	Multivariate analysis Coefficient/cSoef (standard error/SE)					
	Model 1	Model 2	Model 3	Model 4	Model 5	Model 6
	F = 1.62	F = 3.04	F = 1.84	F = 3.04	F = 0.25	F = 0.79
	R^2^ = 0.15	R^2^ = 0.49	R^2^ = 0.66	R^2^ = 0.88	R^2^ = 0.89	R^2^ = 0.92
	P value = 0.173	P value = 0.010	P value = 0.110	P value = 0.018	P value = 0.905	P value = 0.582
**Intercept**	−0.252 (0.37)	−0.184 (0.39)	−0.059 (0.46)	0.337 (0.45)	0.321 (0.56)	0.922 (0.76)
**Age**	0.00 (0.01)	0.00 (0.00)	−0.03 (0.03)	−0.03 (0.02)	−0.02 (0.03)	−0.01 (0.03)
**Gender**						
Female	1 (reference)	1 (reference)	1 (reference)	1 (reference)	1 (reference)	1 (reference)
Male	−0.03 (0.10)	0.01 (0.10)	−0.01 (0.10)	0.09 (0.10)	0.06 (0.12)	0.25 (0.17)
**Level of education**						
Below secondary	1 (reference)	1 (reference)	1 (reference)	1 (reference)	1 (reference)	1 (reference)
Secondary to post-secondary	−0.07 (0.07)	−0.03 (0.06)	−0.04 (0.06)	−0.06 (0.06)	−0.09 (0.10)	−0.17 (0.13)
**Employment status**						
Employed	1 (reference)	1 (reference)	1 (reference)	1 (reference)	1 (reference)	1 (reference)
Unemployed	0.29 (0.14)	0.39 (0.14)*	0.40 (0.14)*	0.17 (0.02)	0.07 (0.30)	0.08 (0.34)
**Country of birth**						
Canadian born	1 (reference)	1 (reference)	1 (reference)	1 (reference)	1 (reference)	1 (reference)
Not Canadian born	−0.09(0.07)	−0.00 (0.07)	0.07 (0.08)	0.07 (0.10)	0.06 (0.12)	0.06 (0.14)
***Index offense characteristics and victim profile***						
**Age at index offense**		−0.01 (0.01)	0.02 (0.03)	0.01 (0.02)	0.01 (0.03)	−0.01 (0.03)
**Intoxication at index offense**						
No		1 (reference)	1 (reference)	1 (reference)	1 (reference)	1 (reference)
Yes		−0.43 (0.11)**	−0.54 (0.011)**	−0.55(0.09)**	−0.49 (0.13)*	−0.64 (0.17)*
**Use of weapon at index offense**						
No		1 (reference)	1 (reference)	1 (reference)	1 (reference)	1 (reference)
Yes		−0.03 (0.08)	−0.07 (0.08)	0.01 (0.07)	0.02 (0.09)	−0.01 (0.10)
**Victim’s gender**						
Male		1 (reference)	1 (reference)	1 (reference)	1 (reference)	1 (reference)
Female		0.05 (0.07)	0.08 (0.07)	0.07 (0.06)	0.08 (0.08)	0.16 (0.09)
**Relationship with victim**						
Known to patient		1 (reference)	1 (reference)	1 (reference)	1 (reference)	1 (reference)
Unknown to patient		−0.04 (0.07)	0.01 (0.07)	−0.07 (0.07)	−0.08 (0.07)	−0.14 (0.09)
**Level of injury to victim**						
Minor/none		1 (reference)	1 (reference)	1 (reference)	1 (reference)	1 (reference)
Requiring hospitalization		0.15 (0.09)	0.19 (0.09)	0.14 (0.09)	0.11 (0.11)	0.19 (0.13)
Resulting in death		0.09 (0.12)	0.14 (0.13)	−0.05 (0.13)	−0.04 (0.14)	−0.13 (0.17)
**Nature of index offense**						
Sexual		1 (reference)	1 (reference)	1 (reference)	1 (reference)	1 (reference)
Non-violent offence		−0.17 (0.10)	−0.08 (0.11)	−0.14 (0.10)	−0.13 (0.12)	−0.20 (0.15)
***Clinical and diagnostic profile***						
**Primary diagnosis**						
Schizophrenia and psychotic spectrum disorder				1 (reference)	1 (reference)	1 (reference)
Bipolar disorder			−0.01 (0.11)	0.13 (0.12)	0.18 (0.14)	0.12 (0.21)
Neurocognitive disorder			0.30 (0.15)	0.24 (0.16)	0.28 (0.19)	0.15 (0.26)
Others			0.28 (0.15)	0.21 (0.15)	0.22 (0.17)	0.37 (0.22)
**Comorbid mental illness**						
No			1 (reference)	1 (reference)	1 (reference)	1 (reference)
Yes			−0.02 (0.07)	−0.08 (0.06)	−0.10 (0.08)	0.00 (0.10)
**Clinical status**						
Out-patient			1 (reference)	1 (reference)	1 (reference)	1 (reference)
In-patient			0.17 (0.07)*	0.20 (0.06)*	0.20 (0.08)*	0.27 (0.08)*
**Length in the forensic psychiatric system (years)**			0.03 (0.03)	0.02 (0.02)	0.01 (0.03)	−0.01 (0.04)
**Capacity to consent for treatment**						
No			1 (reference)	1 (reference)	1 (reference)	1 (reference)
Yes			−0.03 (0.08)	0.00 (0.11)	0.00 (0.15)	0.06 (0.17)
**Treatment compliance (n = 196)**						
Compliant			1 (reference)	1 (reference)	1 (reference)	1 (reference)
Non-compliant			−0.06 (0.12)	−0.10 (0.12)	−0.10 (0.14)	−0.12 (0.34)
***Psychiatric history and behavioral patterns***						
**Age at first admission**				0.00 (0.00)	0.00 (0.00)	0.00 (0.00)
**History of substance use misuse**						
None				1 (reference)	1 (reference)	1 (reference)
Yes, alcohol and other illicit drugs				0.17 (0.08)*	0.17 (0.09)	0.22 (0.11)
Yes, alcohol only				0.24 (0.17)	0.19 (0.22)	0.25 (0.24)
Yes, other illicit drugs only				−0.74 (0.39)	−0.91 (0.50)	−0.76 (0.61)
**History of self-harm**						
No				1 (reference)	1 (reference)	1 (reference)
Yes				−0.07 (0.14)	−0.08 (0.18)	−0.38 (0.25)
**Past violent events to others**						
No				1 (reference)	1 (reference)	1 (reference)
Yes				−0.05 (0.08)	−0.06 (0.09)	−0.01 (0.11)
**Past violent events to objects**						
No				1 (reference)	1 (reference)	1 (reference)
Yes				0.08 (0.09)	0.09 (0.10)	0.20 (0.15)
**Past sexually inappropriate behavior**						
No				1 (reference)	1 (reference)	1 (reference)
Yes				−0.03 (0.07)	−0.01 (0.09)	−0.05 (0.13)
**Previous incapacity to consent for treatment**						
No				1 (reference)	1 (reference)	1 (reference)
Yes				0.01 (0.10)	0.05 (0.14)	−0.03 (0.18)
**Previous poor treatment compliance**						
No				1 (reference)	1 (reference)	1 (reference)
Yes				0.00 (0.10)	0.04 (0.14)	0.09 (0.16)
**Previous response to psychiatric treatment**						
Good				1 (reference)	1 (reference)	1 (reference)
Poor				−0.04 (0.09)	−0.06 (0.11)	−0.07 (0.12)
***Criminal history and legal status***						
**Number of previous convictions**					0.01 (0.03)	−0.03 (0.04)
**History of absconding**						
No					1 (reference)	1 (reference)
Yes					0.03 (0.07)	0.14 (0.10)
**Previous incarceration**						
No					1 (reference)	1 (reference)
Yes					−0.03 (0.08)	−0.04 (0.09)
**Review board status**						
UST					1 (reference)	1 (reference)
NCR					0.09 (0.13)	−0.11 (0.19)
***Recent violent events***						
**Number of recent incidences of verbal aggression**						−0.01 (0.00)
**Number of recent incidences of harm to objects**						0.12 (0.11)
**Number of recent incidences of harm to self**						−0.32 (0.45)
**Number of recent incidences of harm to others**						−0.07 (0.05)
**Number of recent incidences of sexually inappropriate behavior**						0.03 (0.05)

* - p-value < 0.05. ** - p-value < 0.001.

Model 1 included variables from Block 1 and accounted for 15% of the variance in change in risk to public safety (R^2^ = 0.15, p = 0.173). None of these variables showed a statistically significant relationship with changes in risk.

Model 2 incorporated Block 2 variables, increasing the explained variance by 34% (R^2^ = 0.49, p = 0.010). Significant predictors in this model included unemployment (adjusted coefficient [acoef] = 0.39, SE = 0.14, p = 0.008) and intoxication at the time of index offense (acoef = −0.54, SE = 0.11, p < 0.001).

Model 3 added Block 3 variables to Model 2, further increasing the explained variance to 66% (R^2^ = 0.67, p = 0.144). Significant predictors remained similar to Model 2, including intoxication during the index offense (acoef = −0.51, SE = 0.10, p < 0.001) and unemployment (acoef = 0.42, SE = 0.14, p = 0.06). However, only inpatient status (acoef = 0.17, SE = 0.07, p = 0.027) was significantly associated with changes in risk level for public threat among the added variables.

Model 4, which included Block 4 variables, increased the explained variance by an additional 22% (R^2^ = 0.88, p = 0.018). Unemployment was no longer a significant predictor, but a history of abuse of both alcohol and other illicit drugs (acoef = 0.17, SE = 0.08, p = 0.048) was the only significant variable among the added items. Intoxication at the index offense continued to significantly reduce risk (acoef = −0.55, SE = 0.09, p < 0.001), while inpatient status increased risk (acoef = 0.20, SE = 0.06, p = 0.003).

Adding criminal history and legal status variables (Block 5) to Model 4 resulted in a model that accounted for 89% of the variance (R^2^ = 0.89, p = 0.905) in the prediction of change in risk to the public. Intoxication at the index offense remained a significant predictor of lower risk (acoef = −0.49, SE = 0.13, p = 0.003), and inpatient status continued to increase the risk (acoef = 0.20, SE = 0.07, p = 0.010). However, the use of both alcohol and other illicit drugs was no longer significant.

In the final model (Model 6), the addition of Block 6 variables (i.e., recent violent events such as verbal aggression, harm to objects, self, others, and sexually inappropriate behavior) explained an additional 3% of the variance, making the final model account for 92% of the variance in changes in risk (R^2^ = 0.92, p = 0.581). Significant predictors remained similar to Model 5, with intoxication at the index offense (acoef = −0.63, SE = 0.17, p = 0.005) and inpatient status (acoef = 0.27, SE = 0.08, p = 0.010) being key factors.

Overall, Models 2 and 4 were statistically significant predictors, while Model 6 explained the most variance in public threat risk (R^2^ = 0.92). Intercept values increased from −0.252 in Model 1 to 0.922 in Model 6, suggesting the inclusion of impactful variables in the later models.

## Discussion

The present study investigates the changes in the reported level of risk to public safety among older individuals aged 55 years and older within the forensic psychiatry system in Ontario, Canada. The study first investigated the correlation of change in risk levels for a threat to public safety by comparing the past year to the reporting year (2014 and 2015) captured in the ORB database. The results from the present study indicate that certain factors are significantly correlated with a decreased risk of posing a significant threat to public safety. These include being an outpatient and having the capacity to consent to treatment. Individuals receiving outpatient psychiatric care are less likely to pose a high threat to public safety, which is a threshold often required for a disposition with community access and a care plan for community re-integration. This may be attributed to better-controlled mental health conditions and having been tested in the community, and they are maintained with sustained access to community resources. The capacity to consent to treatment is an indicator of an individual’s insight into their illness, and those who are deemed treatment-capable have potentially demonstrated a higher degree of insight and meet the threshold to be independent in treatment decision-making.^27^ In contrast to this finding, those with no capacity to consent to treatment were also associated with a reduction in the risk posed to public safety, possibly due to the effective use of substitute decision-maker programs in maintaining treatment adherence to achieve recovery.

Secondarily, this study utilized a hierarchical regression model to analyze the factors explaining variations in risk tendencies among OAFP. Of the six models included, only Models 2 and 4, which incorporated the characteristics of the index offense with victim characteristics and past psychiatric history with behavioral patterns, respectively, were statistically significant. Model 2 explained the highest variability at 34%, underscoring the importance of circumstances during the index offense in determining risk level. This finding is particularly intriguing as it suggests that the context of the index offense plays a crucial role in future risk assessment and formulation of care plans. Conventionally, individuals in the forensic psychiatric system are rehabilitated and managed based on their current mental status to achieve well-being and mitigate any threat posed to public safety. However, the forensic system still mirrors several aspects of the prison-correctional system, where rehabilitation is influenced by the circumstances surrounding the index offense. This approach may not fully align with recent principles of psychiatric rehabilitation, which emphasize the importance of dynamic individualized treatment plans based on current mental health status and needs.^28^^,^^29^ The significant contribution of past psychiatric-related items and historical criminogenic attributes (Block 4) to the variance in risk (second largest explanation at 22%) highlights the forensic system’s over-reliance on historical factors in predicting risk levels and care plans among older individuals. This reliance raises important questions about the actual importance of these historical clinical factors and the circumstances during the index offense in risk prediction. Our study findings suggest a need for large follow-up studies to determine the relevance or weight of these factors (which are largely static or non-modifiable) in the rehabilitation and planning of care of OAFP. Moreover, elements of potential assessors’ bias in risk assessment need to be critically evaluated. Ensuring that risk judgments are based on dynamic and current items rather than a predominance of historical or contextual factors is crucial for fair and effective rehabilitation.^30^^-^^32^ The integration of dynamic, protective, and modifiable factors into the formulation of risk, disposition, care planning, and management of OAFP aligns with recent gains in traction for nuanced approaches to effective risk management and patient-center care.^24^

Among the predictors in Model 2, the most prominent risk factor for increasing the risk to public safety was unemployed status during the reporting year, a factor that remained significant even in Model 3, but this was not the case when other Blocks were added. Employment has long been recognized as a major protective factor against violence and other risks in the criminal justice system.^33^ However, the significance of this factor in older individuals raises concerns about its predictive validity, although the age distribution of the population analyzed in this study needs to be considered. Since older individuals are often close to retirement age or may be unable to function as effectively as younger individuals in the workforce. Many forensic patients, especially older adults have physical health morbidities, and limited education, often not exceeding post-secondary levels, which further complicates their ability to engage in common forms of employment available to those involved in the criminal justice system.^22^ Also, getting employment is harder for individuals with a criminal record, especially for those who commit serious offenses.^33^ Since gaining meaningful employment, a known protective factor in younger populations, is harder for this group, there is a need for age-appropriate risk prediction factors that do not focus on employment as a contributing factor. Conversely, those who were intoxicated during their index offense had their risk level predicted as reduced, a factor that remained significant even after adding other predictors). This finding may reflect the effectiveness of forensic programs in managing substance use problems, thereby reducing the associated risks among OAFP. Substance use treatment programs within forensic settings have shown positive outcomes in reducing recidivism and the risk of substance misuse post-release.^34^^,^^35^ Interestingly, using both alcohol and other illicit drugs was significant only in Model 4. This may be due to the heightened risk of violence and impulsive activities associated with the use of both addictive substances.

Being an inpatient was significantly associated with a change in risk level following introduction to Model 3 and subsequent models, signifying the key role it plays in predicting change in risk posed by OAFP and their care plan for rehabilitation. The inpatient status may allude to the severity of the mental health conditions in the affected OAFP, warranting hospital admissions and deterring transition or discharge into the community during the period under review. More often, inpatients require intensive treatment to improve their mental health wellbeing and monitoring to mitigate risk. Additionally, inpatients are often predicted to be of higher risk to the public compared with outpatients, and thus, may not have ORB dispositions that allow community living or a change to outpatient status, reinforcing the strong and persistent relationship.^36^^,^^37^

### Limitations

Despite being one of the largest studies involving OAFP and the final study model achieving a high explanatory power (92%), the limited sample size is a shortcoming in this study. Additionally, the retrospective study design resulted in multiple missing values across various variables. Also, there is limited information on the inter-rater reliability or criterion validity of the risk classification used (e.g., low–moderate–high). Finally, the cross-sectional nature of the data limits the ability to draw causal conclusions. Therefore, future longitudinal studies with robust sample sizes are warranted to better understand the predictive nature of the various factors and blocks on changes in risk levels among older adults. Moreover, future research needs to capture inter-rater reliability or criterion validity of risk classification.

## Conclusion and implications

This study model demonstrated a robust explanatory effect size in accounting for the variability in the changes reported in the risk profile of OAFP. It identified supervised care, inpatient status, and intoxication at the time of index offense as critical proxy factors that correlate with the degree of risk to public safety attributed to patients and the determinants of the type of care implemented. These findings highlight the need for resources, policies, and supported care, including inpatient services to manage behavioral issues and support OAFP. Furthermore, the study emphasized the significant influence of historical factors, particularly the circumstances surrounding the index offense and past psychiatric history, in the prediction of future safety concerns and the care plan of OAFP. Implicitly, these findings underscore the need for innovative practices to promote age-appropriate risk assessment and the development of rehabilitation strategies that incorporate dynamic and protective factors. Effective management of OAFP requires interventions tailored to the unique needs and capabilities of individual patients. Future research should focus on developing and validating risk prediction and care planning models for OAFP taking into consideration individual patients’ specific circumstances, dynamic factors, and the need for optimal risk management and care plan strategies. It is also imperative that the reliance of risk formulation and care planning on historical factors among OAFP needs to pave the way for innovative risk management and care models. Such models should involve a better integration of dynamic and protective factors.

## Data Availability

Due to the sensitivity of the population being explored, the datasets will be made available to appropriate academic parties on request from the corresponding author after approval by GAC.

## Data statement

The data has not been previously presented orally or by poster at scientific meetings.
